# Benthic macroinfaunal community structure, resource utilisation and trophic relationships in two Canadian Arctic Archipelago polynyas

**DOI:** 10.1371/journal.pone.0183034

**Published:** 2017-08-29

**Authors:** Anni Mäkelä, Ursula Witte, Philippe Archambault

**Affiliations:** 1 Oceanlab, School of Biological Sciences, University of Aberdeen, Newburgh, Aberdeenshire, United Kingdom; 2 Institut des Sciences de la mer de Rimouski, Université du Québec à Rimouski, Rimouski, Canada; 3 Hopkins Marine Station, Stanford University, Pacific Grove, CA, United States of America; Seoul National University, REPUBLIC OF KOREA

## Abstract

Climate change driven alterations to patterns of Arctic marine primary production, with increasing phytoplankton- and decreasing ice algal production, have the potential to change the resource utilisation and trophic structure of the benthic communities relying on the algae for food. To predict the benthic responses to dietary changes, we studied the macroinfaunal community compositions, and used the faunal δ^13^C and δ^15^N signatures to investigate their main food sources and trophic positions in North Water (NOW) and Lancaster Sound (LS) polynyas in the Canadian Arctic Archipelago. Macroinfaunal density (10 952 ind. m^-2^) and biomass (3190 mg C m^-2^) recorded in NOW were higher than previously found in the Arctic at depths >500m, and significantly higher than in LS (8355 ind. m^-2^ and 2110 mg C m^-2^). This was attributed to higher particulate organic matter fluxes to seafloor in NOW. Polychaetes were significant taxa at both sites in terms of density and biomass, and in addition crustacean density in NOW and bivalve density in LS were high. Facultative filter and surface deposit feeders were highly prevalent at both sites, suggesting feeding plasticity is a successful strategy for accessing different food sources. The macrofaunal δ^13^C signatures reflected the signatures of pelagic particulate organic matter at the sites, and an isotope mixing model confirmed phytoplankton as the main food source for most taxa and feeding guilds. The food web length in LS was longer than in NOW (3.2 vs. 2.8 trophic levels). This was attributed to a larger reliance on reworked organic matter by the benthic community in LS, whereas the high export fluxes at the highly productive NOW resulted in higher rates of selective consumption of fresh algal matter. Despite studies suggesting that loss of ice algae from consumer diets in the Arctic might have a negative impact on the benthos, this study suggests that Arctic macrobenthic communities thrive using phytoplankton as their main food source and should thus be able to cope or even benefit from predicted changes to patterns of primary production.

## Introduction

Climate change is drastically altering the Arctic marine ecosystem, and sea ice cover, the most conspicuous feature of the Arctic Ocean, is being reduced at a rate that could leave the region free of summer sea ice by 2040 [1]. Alongside light availability, summer sea ice is the dominant factor controlling the type, timing and duration of Arctic primary productivity [2], which in turn impacts the functioning and structure of the benthic communities that rely upon the sinking phytodetritus for food [3–5]. Sea ice provides a habitat for ice algae that bloom early in the spring when low levels of light are available, but limits the growth of phytoplankton that require more light and open water to grow [2]. As climate change is rapidly increasing the length of the open water period throughout the Arctic, a significant decrease in ice algal primary production is expected [6,7], whilst the longer phytoplankton growth season is predicted to increase the marine primary production overall [8–11].

Already, areas in the Arctic Ocean with an elongated open water period are eutrophic hotspots with high rates of primary and secondary production [12–16]. The export of particulate organic matter (POM) to the seafloor is higher in these hotspots compared to less productive Arctic waters [17,18], especially during the early spring when zooplankton grazing and growth is limited [19,20]. This tight benthic-pelagic coupling is then generally reflected in the high benthic community biomass at these sites [21–25], although increased water depth weakens the relationship [26]. Information on benthic communities in certain regions, such as the Canadian Arctic Archipelago, is however still scarce [27], making it difficult to assess whether similar environmental controls dictate benthic community composition throughout the Arctic. Especially macrofauna are often underrepresented in benthic community descriptions, despite their significant role in the benthic ecosystem functioning [5,28–30]. Even in North Water Polynya (NOW), one of the most well-studied and most biologically productive ecosystems in the Arctic [31,32], data on benthic macroinfauna is limited in taxonomic detail or spatial extent [24,33–35]. This lack of baseline data makes it difficult to monitor the influence of climate change on the benthic communities as a whole.

Despite studies suggesting that the decreasing summer sea ice cover and increasing overall primary productivity can benefit Arctic benthos [36,37], it is still unclear how the change in the type of primary production will impact the benthic fauna. Despite the lower annual primary production rates compared to phytoplankton [38], ice algae are thought to provide a significant early spring food source for benthic consumers [39,40]. Additionally, ice algal assemblages contain a higher proportion of polyunsaturated fatty acids (PUFA) than phytoplankton [41–43], although specific phytoplankton species can have high PUFA concentrations [44]. As PUFAs are essential for maintenance, reproduction and growth of both pelagic [6,45] and benthic [42,46] organisms, the higher PUFA content is thought to make ice algae superior quality food over phytoplankton [43]. Ice algae have been shown in feeding experiments to be the preferred food item for *Macoma balthica* bivalves and deposit feeders [41,42], but information on the natural contributions of ice algae, phytoplankton and other C sources to the diets of Arctic macrobenthos is mainly available for the shallow (<100 m) communities in the Bering and Chukchi Seas [47–50]. These studies show that organic matter (OM) reworked by bacteria is a significant food source for macrobenthos, with significant macroalgal and terrestrial contributions at near-shore sites [51–58]. The benthic food web structure then reflects the quantity and quality of food available, with longer food webs found in the sites with efficient recycling of refractory material [56]. Whether the benthic macroinfaunal communities in the deep Canadian Arctic Archipelago utilise similar foods and have a similar trophic structure than their shallow water counterparts, is yet unknown.

Stable isotope analysis is a commonly used approach to study resource utilisation and community trophic structures. Stable isotope ratios (^12^C:^13^C and ^14^N:^15^N) can provide insight into trophic relationships and consumer food sources when the traditional methods (eg. gut content analysis, behavioural studies) are inappropriate due to the extremely small size of sediment infauna or other restrictions [59]. In addition, isotope signatures allow the investigation of long term accumulation of OM into tissues, rather than a snapshot of the short-term ingestion. Carbon isotopes can be used to investigate the OM sources used by the benthic consumers, as the change in δ^13^C between the primary producer and the consumer is <1‰ [60–62]. As phytoplankton derived OM is usually more depleted in δ^13^C compared to ice algae derived OM [39,42,63,64], δ^13^C can be used to quantify the reliance of macrofauna on the two types of primary consumers. Additionally, isotope mixing models, utilising both δ^13^C and δ^15^N, can further help to elucidate the proportions of different food items in consumer diets as they allow a degree of variability in both source and consumer signatures to be included [65]. Additionally, δ^15^N shows enrichment of 3.4–3.8‰ per trophic level [60,61,66], and can therefore be used to elucidate the trophic interactions and food web structure within a community.

Previous studies have well illustrated the impacts of overlying water column properties on benthic community standing stock, diet and taxonomic and trophic structures in the shallow Chukchi and Bering Seas [47,50,67], but due to limited research efforts in the Canadian Arctic, our understanding of the benthic communities and their resource utilisation in the region is still elementary. The objectives of this study are thus i) to describe the benthic macroinfaunal community compositions in two contrasting Canadian Arctic Archipelago deep sea sites, North Water Polynya (NOW), the largest and most productive polynya in the northern hemisphere [17,20,39], and the smaller Lancaster Sound Polynya (LS), and ii) to compare the macroinfaunal resource utilisation and trophic relationships using faunal δ^13^C and δ^15^N signatures. The two sampling stations were selected because they are among the deepest sites in the Canadian Arctic [35], but the primary productivity in NOW is significantly higher than in LS [14,68]. Whereas only 3% of the local primary production in NOW is attributed to ice algae, in LS they contribute up to 10% [68–70], making the sites ideal for comparing the impact on quality and quantity of food on the benthos. We hypothesise that the higher surface water productivity at the NOW station supports a higher benthic macroinfaunal density and biomass compared to the LS station, but that less abundant food leads to a more complex benthic food web in LS.

## Methods

### Sampling sites

**Fig 1:**
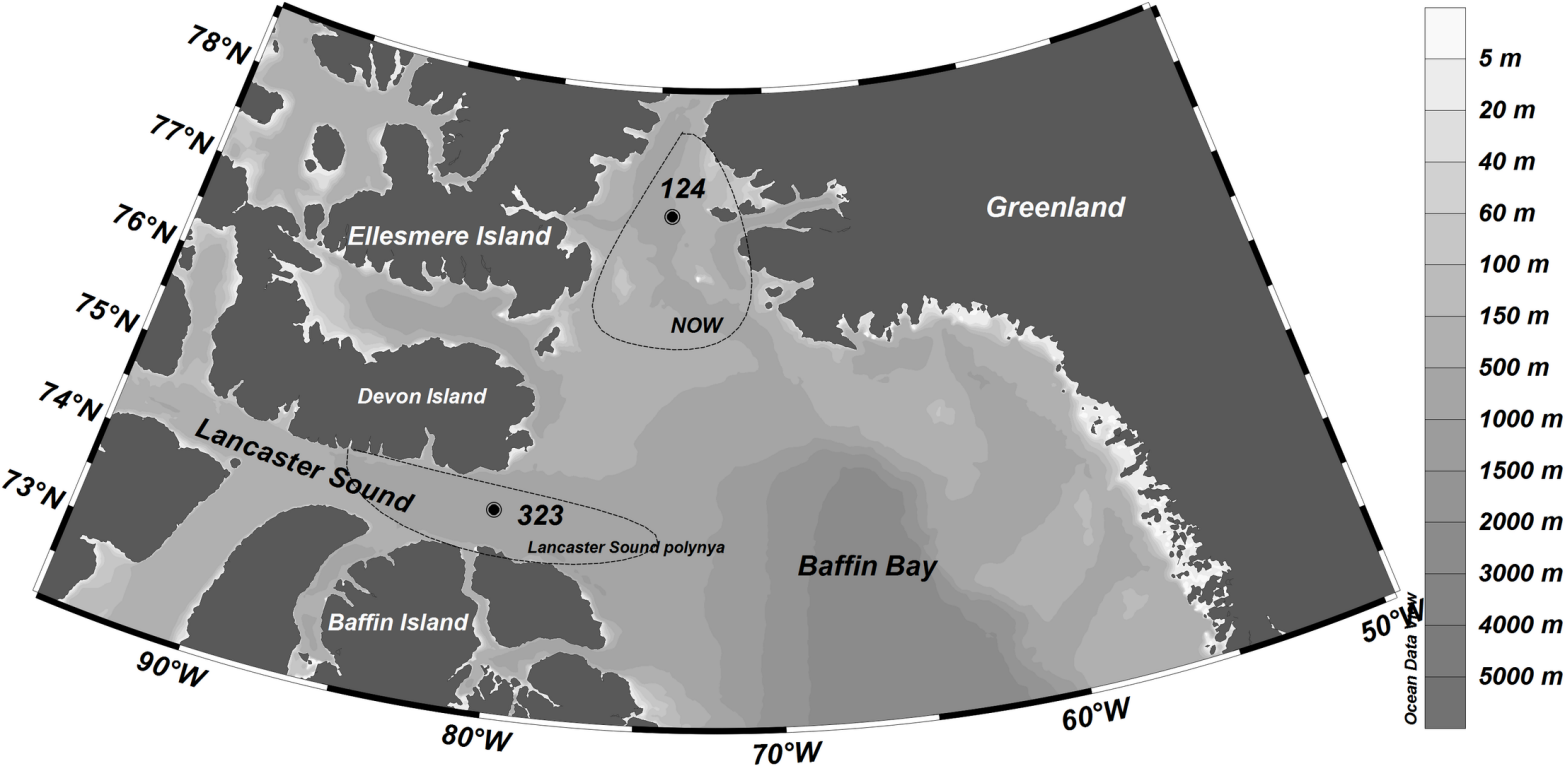
Locations of the sampling sites in North Water Polynya (st. 124) and Lancaster Sound Polynya (st. 323).

Field sampling took place in August 2013 (Table 1) during the ArcticNet 2013 cruise aboard the research icebreaker CCGS Amundsen. The two study locations, North Water Polynya (station 124) and Lancaster Sound Polynya (station 323), are both situated in the western Baffin Bay region in the Canadian High Arctic (Fig 1, Table 1). With a maximum extent of 80 000 km^2^, NOW is regarded as one of the largest and well-studied polynyas in the Northern hemisphere [71] and the largest in the Canadian Arctic [13]. LS and NOW polynyas are among the most biologically productive areas in the Canadian Arctic [13,14,72], with estimated annual new production rates of 60 g C m^-2^ y^-1^ and 254 g C m^-2^ y^-1^, respectively [14,68]. The maximum summer extents of the polynyas have been included in Fig 1 [73]. Peak phytoplankton production in NOW reaches 1.11 g C m^-2^ d^-1^, which is an order of magnitude higher than in adjacent Baffin Bay waters [17]. The peak phytoplankton blooms occur in May-June and July-August time in NOW and LS, respectively [14,17,74]. Both sites had been ice free (<1/10 ice cover) for two weeks before the sampling took place. Ice cover was confirmed from the Canadian Ice Service (CIS) weekly ice charts on eastern Canadian Arctic, available at http://iceweb1.cis.ec.gc.ca/Archive20/page1.xhtml.

**Table 1:** Sampling station, hydrographic and sediment characteristics in Lancaster Sound and North Water Polynya (NOW) in August 2013.

	Lancaster Sound	NOW
ArcticNet 2013 station number	323	124
Date sampled	14.08.2013	27.08.2013
Depth (m)	794	709
Latitude	74°9.41 N	77°20.79 N
Longitude	80°28.32 W	74°17.50 W
Bottom O2 concentration (ml l^-1^)	4.1	5.6
Bottom temperature (°C)	1.2	-0.1
Bottom salinity	34.5	34.4
Sea ice cover (%)	0	0
Sediment OM content (%)	11.3	9.5
Sediment chl-*a* (mg DW m^-2^)	12.9	20.5
Sediment phaeopigments (mg DW m^-2^)	64.5	60.5
Sediment total pigments (mg DW m^-2^)	77.4	81
Chl-*a*/Phaeopigment ratio	0.2	0.34
C/N (w/w, n = 9)	6.55 (±0.07)	6.49 (±0.09)
Surface sediment δ^13^C (n = 3)	-20.0 (±0.7)	-20.6 (±0.7)
Surface sediment δ^15^N (n = 3)	8.3 (±0.3)	7.4 (±0.1)

Field sampling licences were obtained from the Danish Ministry of Foreign Affairs (Survey License #55.GRØNLAND.9 for collection and/or acquisition of biological resources for research purposes in Greenland Waters), United Nations (Consent to Conduct Marine Scientific Research in Areas Under National Jurisdiction of Greenland), Nunavut Research Institute (NRI) (Scientific Research License #0500913N-M), Northwest Territories (Aurora Research Institute Northwest Territories Scientific Research License #15213 and #15271) and Department of Fisheries and Oceans Canada (License to fish for scientific purposes in the waters of the Northwest Territories, Yukon North Slope, and Nunavut Territory #S-13/14-1021-NU).

### Sample collection

Two USNEL box corers (0.25m^2^) were taken from the seafloor at each station to allow for collection of 15 ~10 cm diameter sediment sub-cores. Half of each sub-core was used for macrofauna collection and the other half for phospholipid fatty acid analysis to quantify the sediment bacterial biomass (Mäkelä et al. in prep.). The cores were sliced at 0–5 cm and 5–10 cm sediment horizons, sieved through a 500 μm sieve, and the macrofauna collected was preserved in 4% buffered seawater formalin until analysis. Formalin preservation was used to ensure the small and fragile faunal samples would remain intact to allow for identification to the lowest possible taxonomic level [75]. Data from all 15 cores were used for density, biomass and community composition analysis and samples from 3 cores were used to determine invertebrate tissue δ^13^C and δ^15^N signatures. At each site, water temperature, salinity and dissolved oxygen concentrations were recorded 10 meters above seafloor using a ship board CTD profiler. Additional surface sediment (top 0.5 cm) samples for sediment pigment (chl-*a* and phaeopigment) analysis were also collected from the box corer. Approximately 2 g of the sediment were used for the pigment analysis following the method by Riaux-Gobin & Klein [76]. Sediment samples were stored at -80°C prior to processing.

### Community composition

The macrofauna samples were identified to the lowest possible taxonomic level. Specimens without heads as well as unidentified animal fragments were excluded from the density, but included in the biomass calculations. The specimens were placed in pre-weighed tin cups and dried for 48 h at 60°C, after which the dry mass was recorded. The measurements include shells but not tube structures of polychaetes. Specimens of nematodes and foraminifera were excluded from the biomass and density calculations and the isotope analysis, as these taxa are usually regarded as meiofauna and the majority of the specimens were not retained in the sieve. The community composition was described in terms of total community C and N biomass and density (average from 15 replicates/station), as well as to phylum, family or species level depending on data resolution (pooled accounts from 15 cores/station). The faunal C and N biomass was calculated by multiplying the sample dry mass with the C and N content of each sample, obtained during the isotope ratio determination.

Attribution of feeding guilds was according to the best available sources [33,77,78], and all animals that could be identified to low taxonomic level to allow determination of feeding guild were included in the feeding guild composition description for the sites. The animals were assigned to one of the following: facultative filter feeder-surface deposit feeder (FF/SDF), obligate filter feeder (FF), predator-scavenger-omnivore (P/S), subsurface deposit feeder (SSDF) or obligate surface deposit feeder (SDF).

### δ^13^C and δ^15^N analysis

All fauna from 3 sediment sub-cores were used for the determination of natural abundance isotope signatures, covering majority of the common taxa present at the sites. As the size of individual fauna was extremely small, several individuals of the same taxa within each core were combined together, homogenized and analysed as one sample to ensure enough biomass for the analysis. This is also why complete individuals instead of selected tissues were used in the analysis. The dried invertebrate samples with carbonate structures (echinoderms, crustaceans and bivalves) were acidified in silver cups by adding drops of 1M HCl [79], and allowed to dry without rinsing to ensure no acid soluble proteins or sample biomass was lost [80]. Similarly, samples collected for sediment δ^13^C and δ^15^N analysis were also acidified with 1M HCl. The sediments were allowed to dry between acid addition (10 μl at a time) and the process was continued until bubbling seized to indicate the inorganic carbon had been dissolved. All samples, including acidified samples in silver cups, were encapsulated in tin cups for analysis. The samples were simultaneously analysed for ^13^C-^15^N isotopic signatures due to limited sample biomass. Samples from 0–5 cm and 5–10 cm layers were analysed separately but combined for the core average.

The total C and N concentrations and the δ^13^C and δ^15^N natural abundance isotope ratios of the pooled animal samples were determined using a PDZ Europa ANCA-GSL elemental analyser interfaced to a PDZ Europa 20–20 isotope ratio mass spectrometer (Sercon Ltd., Cheshire, UK). During analysis, the samples were interspersed with several replicates of at least 4 different National Institute of Standards and Technology (NIST) laboratory standards (glutamic acid GLU, Nylon 5, USGS-41 glutamic acid, REF5 FIVER, IsoLife maize, bovine liver), which have previously been calibrated against NIST standard reference materials (IAEA-N1, IAEA-N2, IAEA-N3, USGS-40 and USGS-41). Long term standard deviation for the stable isotope measurements is 0.2‰ for ^13^C and 0.3‰ for ^15^N.

The total carbon and total nitrogen concentrations and the δ^13^C and δ^15^N natural abundance isotope ratios of the sediments were determined using a Flash EA 1112 Series Elemental Analyser connected via a Conflo III to a Delta^Plus^ XP isotope ratio mass spectrometer (all Thermo Finnigan, Bremen, Germany). The isotope ratios were traceable to International Atomic Energy Agency reference materials USGS40 and USGS41 (both L-glutamic acid); certified both for δ^13^C (‰_VPDB_) and δ^15^N (‰_air N2_). The carbon and nitrogen contents of the samples were calculated from the area output of the mass spectrometer calibrated against National Institute of Standards and Technology standard reference material 1547 peach leaves which was analysed with every batch of ten samples. Long term precisions for a quality control standard (milled flour) were: total carbon 40.24 ± 0.29%, δ^13^C -25.4 ± 0.21‰, total nitrogen 1.72 ± 0.035% and ^15^N 0.3672 ± 0.0001 atom % (mean ± SD, n = 200).

Stable isotope values are expressed in the δ notion (‰) relative to the reference material according to the equation:

$$\delta X\left(‰\right)=\left({\text{R}}_{\text{sample}}-{\text{R}}_{\text{reference}}\right)-1\times 1000$$ (1)

Where X = ^15^N or ^13^C, R_sample_ = is the ^13^C:^12^C or ^15^N:^14^N ratio of the sample and R_reference_ is the ^13^C:^12^C or ^15^N:^14^N ratio of the reference material for C or N. The international reference material for C is the Vienna Pee Dee Belemnite (R_VPDB_ = 0.0112372) and for N it is the isotopic ratio of atmospheric N_2_ (R_atmN_ = 0.0036765).

Formalin preservation is known to alter the δ^13^C signatures of fauna, so to correct for the effect of preservation, 1‰ was added to each δ^13^C measurement [81–84]. This 1‰ correction is supported by several methodological papers which show a decrease of 1‰ in sample δ^13^C signatures after formalin preservation [85–91]. The impacts of formalin preservation on δ^15^N signatures has been shown to be insignificant [75,85–87,91,92] and therefore no correction was applied.

The proportion of ice algae and phytoplankton in the macrofaunal diets was determined by using a Bayesian isotope mixing model (Stable Isotope Analysis in R, SIAR) [93], using ice algae and pelagic POM (a proxy for phytoplankton) δ^13^C and δ^15^N values collected by Roy et al. [94] at the same sampling station 323 in LS and another sampling site in NOW in 2011, as well as values reported by Hobson & Welch in LS [66] and Hobson et al. in NOW [39]. The SIAR model is specifically designed to absorb temporal variability in both food source and consumer isotopic signatures, thus making it an ideal tool for examining the contributions of ice algae and phytoplankton to consumer diets when several end member isotopic profiles are available. The model used 1‰ enrichment/trophic level for δ^13^C and 3.8‰ enrichment/trophic level for δ^15^N.

Additionally, the δ^13^C-δ^15^N signatures of consumers were used to plot the Euclidean distances of samples within the sites to calculate two trophic structure metrics. The mean distance of each sampled taxa to the δ^13^C-δ^15^N centroid (CD) was used to compare the trophic separation, and the mean nearest neighbouring distance (NND) of the taxa was calculated to investigate the trophic redundancies of the sites. Larger mean CD values indicate higher trophic separation and smaller NND values indicate higher trophic redundancy. Trophic metrics were calculated using MATLAB R0214a based on the method of Layman et al. [95].

### Trophic level determination

The trophic level (TL) of each sample was calculated from the sample δ^15^N value, assuming an increase of 3.8‰ in the isotope signature per trophic level. The 3.8‰ stepwise enrichment was chosen as it has previously been established for the marine food web in the Lancaster Sound region [66]. There are several baseline measurements that can be used for determining the consumer trophic positions, including primary consumer δ^15^N values [47,55,67,96] and POM [50,58,97] or ice algal [97] signatures. Additionally, the surface sediment bulk δ^15^N signature can be used, which encompasses both the deposited ice algal and phytoplankton food sources [98], making it a better representation of the C and N pool available to benthos compared to surface water POM at the time of sampling [98,99]. Here the *in situ* sediment δ^15^N signatures were used, which we 8.3 ± 0.3‰ and 7.4 ± 0.1‰ in LS and NOW, respectively (Table 1). The consumer trophic levels were thus calculated using the equation

$${\text{TL}}_{\text{consumer}}=\frac{\left(\delta^{15}{\text{N}}_{\text{consumer}}-\delta^{15}{\text{N}}_{\text{sediment}}\right)}{3.8+1}$$ (2)

### Statistical analysis

Differences in density and C biomass between the study sites was tested using an Independent sample T-test and N biomass was tested using Mann-Whitney U test. Differences in δ^13^C and δ^15^N signatures between sites and feeding guilds was tested for SDF, SSDF and P/S, but FF/SDF group was excluded due to small sample size. The δ^13^C signatures were compared using a Two-Way ANOVA test with site (2 levels: LS and NOW) and feeding guild (3 levels: SDF, SSDF and P/S) acting as the two fixed factors being tested for their main effects and the interaction between them. The δ^15^N signatures were tested separately for within station differences between feeding guilds using either a One-Way ANOVA or Kruskal-Wallis test when data residuals were not normally distributed, and then between sites for each feeding guild using either an Independent samples t-test or a Mann-Whitney U test. Deviations from homogeneity of variance assumption were corrected by using the Welch correction, and Games-Howell post hoc test was used when appropriate. The normality of the data residuals was tested using the Shapiro-Wilk test and the homogeneity of residual variance was tested visually. When required, a log10 transformation was applied to ensure the residual normality assumption was fulfilled.

## Results

### Macroinfaunal density and biomass

Approximately 95% of the specimens were recovered from the upper 5 cm of sediment at both stations (Table 2). Additionally, ~90% of biomass in LS and ~75% in NOW was concentrated in the upper sediment layer. For statistical comparison of total density and biomass, the 0–5 cm and 5–10 cm layers were pooled into one 0-10cm layer. An independent samples t-test showed that the macroinfauna density in NOW was significantly higher than in LS (t = 2.351, df = 28, p = 0.026).

**Table 2:** Vertical distribution of sediment macroinfaunal density and macroinfaunal C and N biomass at the North Water Polynya (NOW) and Lancaster Sound (LS) sites. Data are mean ± SE (n = 15). LS biomass calculations excluded 3 large bivalves found in the cores.

Site	Sediment layer (cm)	Density (ind. m^-2^)	Biomass (mg C m^-2^)	Biomass (mg N m^-2^)
**NOW**	0–5	10538 ± 860	2465 ± 203	422 ± 38
	5–10	414 ± 46	725 ± 306	145 ± 72
**LS**	0–5	7949 ± 488	1937 ± 340	441± 81
	5–10	407 ± 99	173 ± 31	39 ± 7

The C biomass at the NOW (Table 2) site was found to be significantly higher than the LS biomass (Independent samples t-test, t = 2.746, df = 26.168, p = 0.011). Differences in N biomass between the sites were analysed using a Mann-Whitney U test and no statistically significant difference was found between the sites (U = 85.000, p = 0.267). In one of the cores in NOW, the presence of one larger Maldanidae polychaete in the 5–10 cm layer contributed 41% and 50% to C and N biomass, but was considered macrofaunal sized and included in the total core estimation. LS had 3 large bivalves, mainly *Bathyarca glacialis* individuals, present in the cores. While these bivalves are classified as infauna, they were regarded as megafauna as they were an order of magnitude greater than all other fauna collected, with the three individuals amounting to twice as much biomass as the other 475 individuals collected at the site combined. These 3 individuals were therefore excluded from the data presented in Table 2 and from the statistical analysis of the macroinfaunal biomass between the sites. They were also excluded from the community biomass description. The total biomass of these 3 large individuals amounted to 3666 mg C m^-2^ and 833 mg N m^-2^, indicating the occasional megafauna can have a large impact on the benthic faunal biomass.

### Community composition–density

Overall 31 family level taxa were identified at both sites, which included 16 polychaetes, 10 crustaceans (10 genera), 3 bivalves and 2 ophiuroids in NOW, and 18 polychaetes, 5 crustaceans (6 genera), 6 bivalves, 1 caudofoveata and 1 sipunculid in LS (Table 3). At both sites polychaetes were significant taxa in terms of macroinfaunal density, with 35% and 43% contribution to the total community at the NOW and LS sites, respectively. Additionally, NOW had a high density of crustaceans (57%), whereas the bivalves contributed 41% to overall community density in LS with the family Thyasiridae making up most of the density and biomass. The polychaete species *Prionospio cirrifera* and the cumacean *Eudorellopsis integra* were especially abundant, with *P*. *cirrifera* making up 8% and 13% of the whole community density in NOW and LS, and *E*. *integra* contributed 45% to macroinfaunal density in NOW. The majority of the animals (including the highly abundant Spionidae polychaetes and Leuconidae cumaceans) at both sites were identified as FF/SDF. This feeding guild therefore dominated density at both sites.

**Table 3:** Relative density and C and N biomass of sediment macroinfauna in North Water Polynya (NOW) and Lancaster Sound (LS) sampling sites. The data are presented without the 3 megafaunal *Bathyarca glacialis* bivalves in LS.

	North Water			Lancaster Sound			
Taxa	Density (%)	C biomass (%)	N biomass (%)	Density (%)	C biomass (%)	N biomass (%)	Feeding guild
**Nemertea**	< 1	4.0	4.0	< 1	< 1	< 1	
**Platyhelminthes**				< 1	< 1	< 1	
**Porifera**				< 1	< 1	< 1	FF
**Sipuncula**	< 1	< 1	< 1	5.1	1.0	1.1	
Golfingiidae				< 1	< 1	< 1	SDF
**Ascidiacea**	< 1	< 1	< 1				FF
**Actiniaria**	< 1	2.2	2.2				P/S
**Echinodermata total**	**1.1**	**1.6**	**2.0**	**1.3**	**< 1**	**< 1**	** **
Asteroidea	< 1	< 1	< 1	< 1	< 1	< 1	
Ophiuroidea	< 1	< 1	< 1	< 1	< 1	< 1	
Ophiurida	< 1	< 1	< 1	< 1	< 1	< 1	FF/SDF
Amphiuridae	< 1	< 1	< 1				FF/SDF
Ophiolepididae	< 1	< 1	< 1				FF/SDF
**Scaphopoda**				< 1	< 1	< 1	
**Caudofoveata**: *Chaetoderma sp*.				< 1	< 1	< 1	P/S
**Bivalvia total**	**4.8**	**9.6**	**10.1**	**40.8**	**7.9**	**7.2**	** **
unidentified juveniles	3.7	5.9	6.2	19.8	3.0	2.7	
Arcidae				< 1	< 1	< 1	FF
Mytilidae	< 1	< 1	< 1				FF
*Cuspidaria* sp.				< 1	< 1	< 1	P/S
*Nucula* sp.	< 1	< 1	< 1				FF/SDF
Pectinidae				< 1	1.5	1.4	FF
Thyasiridae	< 1	< 1	< 1	20.2	3.2	2.8	FF/SDF
*Thyasira* sp.	< 1	2.7	2.9				FF/SDF
Yoldiidae				< 1	< 1	< 1	FF/SDF
**Crustacea total**	**57.3**	**10.5**	**7.4**	**7.6**	**3.8**	**2.6**	** **
**Amphipoda**				< 1	< 1	< 1	
Amphipoda				< 1	< 1	< 1	
Ampeliscidae	< 1	< 1	< 1				FF
Lysianassidae	< 1	< 1	< 1				P/S
*Arrhis* sp.	< 1	< 1	< 1	< 1	< 1	< 1	FF/SDF
*Aceroides latipes*							P/S
*Monoculodes* sp.	< 1	< 1	< 1				P/S
**Cumacea**				1.1	< 1	< 1	
Diastylidae	1.3	< 1	< 1	< 1	< 1	< 1	FF/SDF
*Diastylis* spp.	1.4	< 1	< 1	< 1	1.0	< 1	FF/SDF
*Diastylis rathkei*				< 1	< 1	< 1	FF/SDF
*Ektonodiastylis nimia*				< 1	< 1	< 1	FF/SDF
*Leptostylis* spp.				1.7	< 1	< 1	FF/SDF
Leuconidae	< 1	< 1	< 1	< 1	< 1	< 1	FF/SDF
*Eudorella* sp.	< 1	< 1	< 1	< 1	< 1	< 1	FF/SDF
*Eudorellopsis deformis*	< 1	< 1	< 1				FF/SDF
*Eudorellopsis integra*	45.4	7.2	4.9				FF/SDF
*Eudorellopsis* spp.	1.9	< 1	< 1				FF/SDF
*Leucon* spp.	< 1	< 1	< 1	1.1	< 1	< 1	FF/SDF
**Isopoda**							
Desmosomatidae	1.6	< 1	< 1	< 1	< 1	< 1	
*Desmosoma* sp.	< 1	< 1	< 1				SSDF
*Eugerda* sp.	< 1	< 1	< 1				SSDF
**Tanaidacea**							
Akanthophoreidae	< 1	< 1	< 1				
*Leptognathia* sp.	< 1	< 1	< 1				FF/SDF
Pseudotanaidae	< 1	< 1	< 1				
Sphyrapodidae	< 1	< 1	< 1				
*Pseudosphyrapus* sp.	1.8	< 1	< 1				P/S
**Polychaeta total**	**35.3**	**71.9**	**74.0**	**42.7**	**86.4**	**88.2**	** **
unidentified fragments		14.8	13.8		12.9	13.0	
Acrocirridae				< 1	< 1	< 1	FF/SDF
Ampharetidae				1.1	6.5	5.7	SDF
*Ampharete* sp.	< 1	< 1	< 1	< 1	< 1	< 1	SDF
Capitellidae	< 1	< 1	< 1	< 1	< 1	< 1	
*Spiochaetopterus (typicus*?*)*				< 1	< 1	< 1	FF/SDF
Cirratulidae	2.4	3.9	3.8				
*Chaetozone* spp.	3.7	9.0	8.0	< 1	< 1	< 1	FF/SDF
*Cossura pygodactylata*	< 1	< 1	< 1				SSDF
*Cossura* spp.	8.7	2.6	1.0	< 1	< 1	< 1	SSDF
Flabelligeridae				< 1	< 1	< 1	
*Pherusa* sp.				< 1	< 1	< 1	FF/SDF
Lumbrineridae	0.0	1.3	1.4	< 1	1.4	1.5	P/S
*Abyssoninoe hibernica*				< 1	< 1	< 1	P/S
*Abyssoninoe* spp.				< 1	< 1	< 1	P/S
*Lumbrineris mixochaeta*	< 1	< 1	< 1	< 1	< 1	< 1	P/S
*Scoletoma fragilis*	< 1	< 1	< 1				P/S
*Scoletoma* spp.	< 1	1.1	1.1	< 1	< 1	< 1	P/S
Maldanidae	0.0	16.2	21.1	3.4	6.5	6.4	
*Asychis* spp.				2.3	5.3	5.1	SSDF
*Clymenura polaris*	< 1	< 1	< 1				FF
*Maldane arctica*				< 1	< 1	< 1	SSDF
*Maldane sarsi*				1.1	1.0	< 1	SSDF
*Maldane* spp.				1.5	1.2	1.2	SSDF
Nephtyidae	< 1	< 1	< 1	< 1	< 1	< 1	P/S
*Aglaophamus malmgreni*				1.1	10.3	11.1	P/S
*Aglaophamus* sp.				< 1	15.3	16.6	P/S
*Bipalponephtys neotena*	< 1	< 1	< 1				P/S
*Micronephthys minuta*	< 1	< 1	< 1				P/S
*Micronephthys* sp.	< 1	< 1	< 1				P/S
*Nephtys* sp.				< 1	< 1	< 1	P/S
*Nereis* sp.				< 1	< 1	< 1	P/S
Opheliidae				< 1	< 1	< 1	
*Ophelina cylindricaudata*	< 1	< 1	< 1				SSDF
*Scoloplos group armiger*	< 1	< 1	< 1	< 1	< 1	< 1	SSDF
Oweniidae	< 1	< 1	< 1				
Paraonidae	< 1	< 1	< 1				
*Aricidea nolani*	< 1	< 1	< 1				SDF
*Aricidea* spp.	< 1	< 1	< 1				SDF
*Eteone* sp.	< 1	< 1	< 1				P/S
Sabellidae				< 1	< 1	< 1	
*Chone* spp.				< 1	< 1	< 1	FF
*Euchone incolor*	< 1	< 1	< 1				FF
*Euchone* spp.	< 1	< 1	< 1				FF
Sphaerodoridae				< 1	< 1	< 1	
*Sphaerodoridium minutum*				< 1	< 1	< 1	SSDF
*Sphaerodoropsis* spp.				< 1	< 1	< 1	SSDF
*Sphaerodorum* spp.				< 1	< 1	< 1	SSDF
Spionidae	< 1	1.6	1.7	1.3	7.3	7.5	
*Prionospio cirrifera*	7.9	8.8	8.8	12.6	9.4	9.6	FF/SDF
*Prionospio* sp.	4.2	5.2	5.3	8.2	3.8	3.9	FF/SDF
Syllidae				< 1	< 1	< 1	
*Anguillosyllis* spp.				< 1	< 1	< 1	SDF
*Streptospinigera niuqtuut*				< 1	< 1	< 1	
*Syllides* sp.	< 1	< 1	< 1				P/S
Terebellidae		3.9	4.6	< 1	< 1	< 1	

Feeding guild determinations are as follows: P/S = predator/scavenger, SDF = surface deposit feeder, SSDF = subsurface deposit feeder, FF/SDF = facultative filter/surface deposit feeder, FF = obligate filter feeder.

### Community composition–biomass

In terms of C and N biomass, polychaetes were the dominant taxa in both NOW and LS, as they contributed over 70% and 80% to the total community biomass (Table 3). The significance of crustaceans and bivalves decreased when biomass is considered, largely due to their small size compared to polychaetes. Again *Prionospio cirrifera* contributing ~ 9% to the total community biomass at the NOW and LS. Additionally, larger polychaete groups, such as maldanids, nephtyids and cirratulids contributed significantly to the overall maroinfaunal biomass.

### Macroinfauna resource utilisation

**Fig 2:**
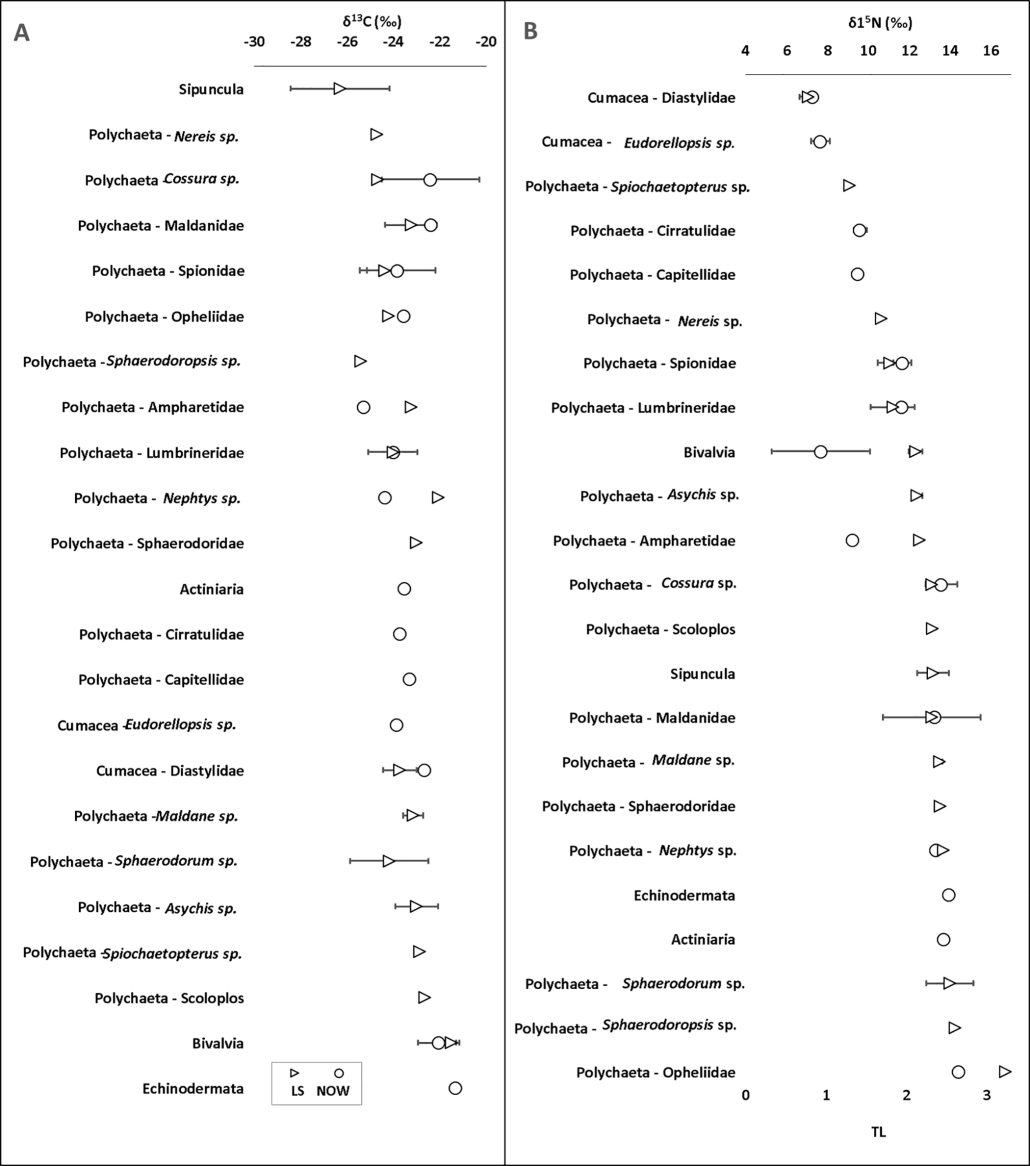
Average A) δ^13^C and B) δ^15^N signatures (± SD) and trophic level (TL) determinations of benthic macroinfauna collected at the North Water Polynya (NOW) and Lancaster Sound (LS) sampling sites.

**Fig 3:**
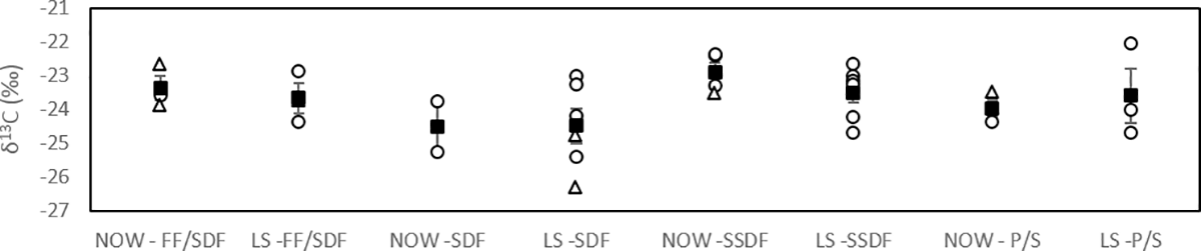
Macroinfaunal feeding guild δ^13^C signatures in North Water Polynya (NOW) and Lancaster Sound (LS). Individual taxa are marked with open symbols and feeding guild averages (± SE) with filled symbols. Circles represent polychaete groups and triangles other taxa. Every data point represents different taxa assigned to that feeding guild. P/S = predator/scavenger, SDF = surface deposit feeder, SSDF = subsurface deposit feeder, FF/SDF = facultative filter/surface deposit feeder, FF = obligate filter feeder.

The average macroinfaunal δ^13^C signatures in NOW and LS were -23.4 ± 1.0‰ (range: -25.3‰ to -21.3‰) and -23.7 ± 1.2‰ (range: -26.3‰ to -21.5‰), respectively (Fig 2A). The average feeding guild δ^13^C signatures (Fig 3) were not significantly different between the stations (F = 0.023, df = 1, p = 0.880) or feeding guild (F = 3.254, df = 2, p = 0.062), nor was a significant interaction between the two overserved (F = 0.453, df = 2, p = 0.643). The proportion of pelagic POM in the diets of most consumers in NOW was high, but *Cossura* sp. and Cirratulidae polychaetes as well as bivalves have a more even contribution ice algae and phytoplankton in their diets (S1 Table). Similarly, in LS, a high proportion of pelagic POM was found in the diets of most taxa, but also an evenly mixed diet of ice algae and POM is seen in 5 polychaete taxa and bivalves, especially in Spionidae polychaetes and bivalves where the contribution of ice algae was slightly higher than that of POM. Additionally, all feeding guilds at both stations had a high proportion of pelagic POM in their diets, with ranges between 0.591 (SDF in NOW) and 0.795 (SSDF in LS).

### Community trophic structure

**Fig 4:**
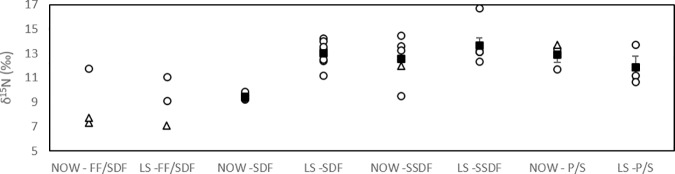
Macroinfaunal feeding guild δ^15^N signatures in North Water Polynya (NOW) and Lancaster Sound (LS). Individual taxa are marked with open symbols and feeding guild averages (± SE) with filled symbols. Circles represent polychaete groups and triangles other taxa. Every data point represents different taxa assigned to this feeding guild. P/S = predator/scavenger, SDF = surface deposit feeder, SSDF = subsurface deposit feeder, FF/SDF = facultative filter/surface deposit feeder, FF = obligate filter feeder. For FF/SDF the average is not shown to illustrate the switching between feeding modes depending on the taxa.

The macroinfaunal δ^15^N values ranged from 7.3 to 14.4‰ in NOW and 7.0 to 16.7‰ in LS (Fig 2B). The faunal trophic levels were 1.0–2.8 (NOW) and 0.7–3.2 (LS) (Fig 2B), with an average TL of 2.0 ± 0.7 in NOW and 2.1 ± 0.6 in LS. The majority of the taxa were therefore secondary consumers. The differences in δ^15^N signatures of feeding guilds (Fig 4) were tested separately for each station due to the non-normal nature of the data residuals for SSDF in LS, which omitted the use of a Two-way ANOVA test. The statistical analysis revealed no significant difference in the signatures between feeding guilds in LS (X_2_ = 1.308, df = 2, p = 0.520) but in NOW there were significant differences (F = 16.226, df = 2, p = 0.012). A Games-Howell post-hoc test showed that SDF signature was significantly lower than SSDF signature (p = 0.047) and P/S signature (p = 0.047). Additionally, a pairwise comparison of each feeding guild signature between the sites showed that the SDF signature in NOW was significantly lower than in LS (t = -8.527, df = 7.996, p <0.001). There were no differences between the sites in P/S (t = 0.964, df = 3.430, p = 0.398) or SSDF (U = 14.000, p = 0.931) signatures. The NND, representation of trophic redundancy, of consumers at the NOW site was 2.78 and at LS it was 2.45. The calculated CDs, or trophic diversities, were 2.64 and 1.98 in NOW and LS, respectively.

## Discussion

### Macroinfaunal density and biomass

Due to the known correlation between surface water productivity and benthic community abundance and biomass [100], we originally hypothesised that the benthic macroinfaunal density and biomass at the NOW sampling station, located in one of the most productive regions in the Arctic Ocean, would be higher than in the LS sampling station. This was proven correct on both accounts, but additionally the biomass and density recorded at the two stations illustrate that both sites are among the most significant Arctic benthic macroinfaunal community hotspots. Comparisons of macrofaunal biomass and density between studies is challenging due to the variety of sampling methods used (trawls, cores), mesh sizes used for sample sorting (usually a range of 250–1000 μm), differences in biomass reporting (wet mass, dry mass, C mass and conversion factors between them) and depths investigated. A switch from 400 μm to 500 μm mesh size can increase macrofauna density by 3% [101], so measurements of macrofaunal density should be compared with caution. Despite of this, the density of macroinfauna recorded at the NOW sampling station exceeds previous records for depths > 500 m from the Arctic Ocean, and measurements from LS are close to the highest previous measurements ([102,103], and references therein). For LS and north-western Baffin Bay, previous measurements using a 1 mm mesh size sieve suggested density of 857 and 1222 ind m^-2^ for depths > 500 m and 4564 and 5502 ind. m^-2^ for < 100m [24], which is less than reported here, but some of the discrepancy is likely due to differences in sampling methodology. Macrofaunal biomass in the Arctic at > 500 m water depth is usually < 500 mg C m^-2^ [100,104–109]. Some exceptions do exist, notably > 2800 mg C m^-2^ reported at depths of 2950 m in Nansen Basin [106], whereas in north western Baffin Bay, close to our sampling sites, an average biomass of > 1500 mg C m^-2^ at depths of 751–1100 m was recorded [24]. The biomasses measured in NOW and LS during this study are therefore higher than could be expected at depths > 500 m, and the > 3000 mg C m^-2^ recorded at NOW exceeds previous measurements in the Arctic deep sea. In comparison, Roy et al. [25] recorded the epibenthic megafaunal density and biomass at LS and NOW to be < 1–16 ind. m^-2^ and 250–1430 mg C m^-2^ (converted assuming C biomass is 5.6–6.3% of wet biomass [110,111]). The benthic macroinfaunal biomass thus clearly exceeds the megafaunal biomass at both sites, highlighting their dominance in forming the deep sea benthic communities.

Historically, LS benthic biomass has been considered among the highest recorded in the Arctic [24], but our results show that the macroinfaunal biomass in NOW is significantly higher still. Depth is generally the main variable controlling macrofaunal density and biomass locally [104–106,112,113], mainly due to its role as a proxy for food availability [114]. In the Canadian Arctic, water depth is however generally not a good indicator of benthic biomass, due to meso-scale processes enhancing the deep sea food supply at locations like polynyas [25]. Similarly to polynyas, marginal ice zones can enhance the local primary productivity and export of OM to the seafloor, whereas perennially ice covered regions and open sea areas support a lower benthic standing stock [103,115–117]. The sediment pigment concentration and C:N ratio, indicators of fresh labile OM input, are similarly significant predictors of benthic standing stock [3,21,22,118]. A low C:N ratio (< 7) in surface sediments is a good indicator of deposition of ungrazed OM with a high nutritional value to the seafloor [21,118,119], and as the C:N ratio at both study sites was ~6.5 at the time of sampling, the OM available to the benthos in NOW and LS was of high quality. Additionally in the Arctic, the length of the ice covered period is a good predictor of the benthic community biomass [36,120], as the longer open water period enables higher rates of primary production, and thus increased food availability to the benthos. In addition to the vast Bering shelf and Chukchi Sea green belts, NOW is the most productive single area in the Arctic [12], and the peak primary production rates in NOW, as well as the Bering Strait, are comparable to the primary production in eutrophic temperate waters [14]. Less is known about the productivity patterns in LS, but the area is also considered to have eutrophic conditions and productivity is thought to be high [9,16,121]. Albeit limited in spatial extent, the examples from the two sampling sites here suggests that despite the water depth, the high local primary productivity in NOW and LS is well reflected in the benthic community standing stock, and the two sites are among the most significant macroinfaunal hotspots recorded in the Arctic.

### Macroinfaunal community structure

In the sediments of the Arctic Ocean, the macroinfaunal community composition is dominated by polychaetes, crustaceans and bivalves in terms of species numbers, biomass and density [24,104,112,113,118], and the two study sites here are no exception. A total of 21 polychaete families were identified from our two sampling stations, which appears high compared to previous studies from the Arctic. Piepenburg et al. [22] recorded 30 polychaete families from 40 stations in Northeast Water Polynya, Grebmeier et al. [5] reported 21 dominant families from 49 station in Bering and Chukchi Seas, and Coyle et al. [122] 41 families from 215 stations in Bering Sea shelf. A previous inventory of macrofauna in NOW found 26 families in total of 4 sampling sites [123]. It is therefore surprising that only 2 sampling stations accumulate 21 families, but shows that the sampling efforts here produced results that are comparable to previous efforts in the region [123]. It has previously been shown that dominant polychaete families generally consist of 1 to 2 species, and therefore family level identification can be considered sufficient for community analysis [5]. Indeed, we found the dominance of a single genus or species for the most dominant families. Especially abundant was the species *Prionospio cirrifera*, which accounted for 8–13% of the total macroinfaunal density in NOW and LS. The faunal community structure recorded in NOW during this study is very similar to that recorded by Lalande [123], who also found *Prionospio cirrifera* to be the dominant species alongside *Cossura longocirrata* and *Chaetozone setosa*, both common genera here too. Additionally *Thyasira* bivalves and *Eudorellopsis integra* cumaceans were common at both studies. The community here is thus very comparable to the previous records and shows very little change in the community structure over the past two decades.

The most noticeable difference between the NOW and LS community compositions was the dominance of bivalves in LS and crustaceans in NOW. High primary production combined with high current speeds in LS enables effective filter feeding, which has been thought to be the contributing factor to the high bivalve biomass and density in LS [24]. As the currents get weaker in northern Baffin Bay, the biomass of filter feeding bivalves decreases in favour of deposit feeding crustaceans. Cumaceans, which were very common in NOW, have not received much attention in benthic macrofaunal community descriptions, but have been documented as the dominant macrofauna in the Greenland [124] and Kara Seas [125], as well as being the dominant crustacean group in Northeast Water Polynya [126]. *Eudorellopsis integra* is the most abundant species in NOW in our study as well as one of the NOW stations sampled by Lalande [123]. Outside of this, only a few records of it exist for the Arctic [33,78,127]. This species however seems to be one of the most characteristic species in NOW. It is also noteworthy that despite the dominance of the two species, *E*. *integra* and *P*. *cirrifera*, the overall taxonomic richness remains high. Previous Arctic macrofaunal studies have generally observed low species richness at sites where abundant food supports high benthic standing stock (ie. dominance of a single or few species), whereas in more food limited stations the faunal abundance is low but taxonomic evenness higher [5,36,128]. Both sites here, however, appear to not only be hotspots for macroinfaunal density and biomass, but also for taxonomic richness.

Finally, regardless of taxonomic composition, the macrofaunal density and biomass at both sites was dominated by facultative filter and surface deposit feeders. This is not surprising as facultative feeders generally tend to make up a large proportion of benthic faunal biomass throughout the Arctic sediments [5,56,129,130]. In this study, the three most dominant taxa overall, the polychaete *Prionospio cirrifera*, crustacean *Eudorellopsis integra* and the bivalve family Thyasiridae are all classified as FF/SDF. The dominance of selective FF/SDF may be a response to a varying quantity and type of OM the benthos receives, where refractory material is supplemented by sporadic pulses of large amounts of fresh food [56], and selectively feeding on available high quality food is an advantage in the highly seasonal environment.

### Macroinfaunal food sources

Bottom ice δ^13^C signatures recorded in NOW have previously been shown to be more enriched than those of pelagic POM, with a range of -14.5 to -17.7‰ for ice algae, and -22.0 to -27.6‰ for POM [39,70,94]. In LS the difference was smaller but still existent, with ice algae signature of -20.7‰, and pelagic POM values in the range of -21.6 to -24.9‰ [66,94]. The low macroinfaunal δ^13^C enrichments in LS and NOW are therefore in agreement with the previous POM measurements above, suggesting macrofaunal dependence on phytoplankton as the main food source at both sites. Additionally, all feeding guilds had similar δ^13^C signatures at both sites, indicating the diets of fauna collected were not feeding guild dependent. The utilisation of phytoplankton food was confirmed by the isotope mixing model for most taxa investigated, but additionally certain taxa, such as bivalves, utilised a mixed diet of both ice algae and phytoplankton at both sites. No taxa in NOW had a higher proportion of ice algae than phytoplankton in their diets, and only 2 (spionids and pooled bivalves) out of the 18 taxa exhibited a slightly higher ice algae dietary contribution in LS. Spionids and bivalves, however, had the opposite dietary contributions in NOW, illustrating how the fauna can change their diet as a response to changing environmental conditions. Similar environmentally driven dietary plasticity has also been reported for other Arctic benthic species [26,131], suggesting that dietary flexibility is common for Arctic benthic fauna. Interestingly, several studies on shallow [48–50,132] and deep sea [56,57] sediment communities have concluded, based on the highly enriched consumer δ^13^C signatures, that the Arctic benthic macrofauna mainly utilise OM degraded by bacteria as their main food source. In this study, the consumer signatures were not unusually enriched compared to the proposed C sources, suggesting degraded material was not the main source of food for the fauna at LS and NOW. This probably reflects the high export of fresh OM to the seafloor in the polynya sites, as macrobenthos at eutrophic sites tend to rely on fresh OM more compared to their counterparts in less productive waters [47,63]. Additionally, contrary to our findings that show macrobenthos mainly feed on phytoplankton, studies looking at the megabenthic C sources in NOW and LS have suggested that ice algae are a major food item for the larger invertebrates [39,94]. This discrepancy is possibly related to the physical limitations of the two size classes: large megafauna with great mobility are able to reach patchy aggregations of ice algae falling on the seafloor [112,133], whereas macroinfauna with limited mobility are more restricted to food in their immediate vicinity. Indeed, Boetius et al. [134] observed megafauna exploiting ice algal aggregations on the Arctic deep sea floor, whereas macrofauna did not utilise the food patches. A real difference in the resource utilisation of the two faunal size classes may therefore exist, and as macrofauna appear less dependent on the ice algal food than the larger consumers, they may be less vulnerable to future changes in the food supply.

It should be noted, however, that the sampling for this study was conducted during the summer open water season. Despite isotope analysis being useful in determining especially the long term resource utilisation, compared to the snapshot traditional stomach content analysis provides [66], some seasonal bias might impact the results. Kaufman et al. [38] experimentally illustrated that it can take up to 4 weeks for the diet switch from isotopically heavy ice algae to lighter phytoplankton to be reflected in the tissues of the sympagic amphipod *Onisimus litoralis* during the Arctic spring. The switch from lighter to heavier isotope signature occurs more slowly. While the tissue δ^13^C turnover times of most fauna have not been investigated in detail, making it difficult to estimate how long it takes for dietary changes to be reflected in the consumer signatures, previous seasonal studies show that the isotopic signature of the main food source of benthos persists in the consumer tissues throughout the year [58]. Therefore, it is unlikely that the overall contribution of ice algae to the consumer diets would be greatly underestimated in this study.

### Trophic interactions

Some effort has been made to construct the entire marine food web in NOW [39,70] and LS [66], but where benthos were included, they were mainly represented by megafaunal taxa, which reached TL 3.6–3.8 [39,66]. Dominant macrofaunal groups such as polychaetes were nearly completely neglected. The present study thus provides the first account of the benthic macroinfaunal food web at NOW and LS. Our original hypothesis was that the lower OM flux in LS would results in a larger reliance on reworked OM for food, and consequently the consumers would occupy higher trophic levels compared to the consumers in NOW. Indeed, in LS the food web was longer than in NOW, with 3.2 and 2.8 TLs, respectively. The food webs at both sites are however shorter than what most other studies have found: where both mega- and macrofaunal taxa were included, 4 TLs have been reported in Beaufort Sea [53,96,98], Chukchi Sea [67] and Svalbard Archipelago [55,58]. Also the megafaunal [23,94] and macrofaunal [56] food webs alone have been shown to involve 4 TLs, whereas in the extremely food limited HAUSGARTEN deep sea observatory up to 5 TLs were found with both faunal size classes included [57]. Simple food webs are found at sites with abundant fresh food and tight benthic-pelagic coupling [96], but benthic food webs consisting of 2–3 trophic levels, like in this study, are rare in the Arctic and have mainly been found in productive polynyas [39,63,66]. High abundance of fresh food also explains why many of the taxa in this study belong to TL 1. Additionally, Diastylidae cumaceans in LS has such a low δ^15^N signature, that the group was assigned TL 0.6 and in general all cumaceans had TL ≤ 1.1 This finding is remarkably consistent with other studies that have also found the trophic position of this family to be very low, close to or below the food source baseline TL of 1 [56–58,96]. This is generally credited to use of extremely fresh OM with low δ^15^N signature [56], fractionates the isotopes differently than other taxa [58] and/or the high exoskeleton-tissue ratio can underestimate the trophic levels during analysis [135].

The distribution of taxa along the trophic continuum was also different between the sites. Knowing how many taxa place in each trophic level is a useful metric for investigating food web structure, as the relative proportion of taxa on low trophic levels (ie. primary consumers) reflects the tightness of coupling between the pelagic food source and benthic consumers [47]. In NOW, the direct utilisation of pelagic OM resulted in an even number of taxa on TL1 and TL2, with 6 and 8 taxa identified as each. In LS, only 4 taxa placed in TL1, whereas 12 belonged to TL2 and one to TL3. While all taxa at both sites seem to utilise fresh food, as indicated by a relatively simple food web, the relative dominance of higher consumers at LS indicates a looser benthic-pelagic coupling, and some utilisation of recycled OM for food. The food limitation is however not as dramatic compared to the deep Canada Basin, where majority of the consumers clustered on the third and fourth TL due to high utilisation of reworked OM [56].

Additionally, the trophic diversity and redundancy analyses suggest that the higher abundance of fresh food at NOW promotes more selective and specialised feeding compared to LS. The community trophic diversity was higher at NOW than in LS, indicating a clearer overall trophic niche separation within the benthic community [95]. Consequently, the trophic redundancy in LS was higher, as most consumers appear to be generalist feeders utilising a higher proportion of recycled OM, compared to more selective feeders in NOW. Although most taxa found at both sites generally had similar δ^15^N signatures, indicating a similar trophic position, certain taxa were notable exceptions that illustrate the differences suggested by the trophic structure parameter calculations. For instance, the δ^15^N signature of the surface deposit feeding polychaete family Ampharetidae is 9.2‰ (or TL 1.5) in NOW and 12.5‰ (or TL 2.1) in LS. Similarly, the SSDF polychaete family Opheliidae showed a difference in the signatures, with 14.4‰ (TL 2.8) and 16.7‰ (TL 3.2) in NOW and LS, respectively. This difference in the δ^15^N signatures suggests selective feeding of fresh algae in NOW and a greater reliance on reworked OM matter in LS [56,136]. The most interesting discrepancy between the sites is however observed in the signatures of bivalves. In NOW the average δ^15^N signature of bivalves was 7.7‰ (TL 1.1) and in LS it was 12.3‰ (TL 2.1), indicating that in NOW they are mainly primary consumers and in LS they occupy a higher trophic level. Due to the juvenile state of some of the bivalves collected, the individuals were extremely small and samples from different families had to be pooled together to ensure sufficient biomass for isotope analysis, especially in NOW where bivalves were rare. Therefore, the taxonomic resolution in NOW is low. Most of the bivalves in LS were however members of the family Thyasiridae, which are classified as FF/SDF. For Arctic deep sea benthos the enrichment generally differs between feeding guilds, with the δ^15^N signatures of deposit feeders < suspension feeders < predator/scavengers [57]. Therefore the bivalve community in NOW probably mainly consist of deposit feeders, whereas the facultative FF/SDF bivalves in LS seem to utilise more filtering, probably as a response to elevated current speed, which promotes filter feeding at the site [24]. The different environmental conditions could therefore influence the trophic positions of the bivalves at the two sites.

Other studies on Arctic benthos have found that the consumer δ^15^N signatures were in general agreement with their assumed feeding behaviour [57,63], but here the faunal tissue δ^15^N signatures did not always reflect their feeding guild determination. In LS, the δ^15^N signatures of different feeding guilds could not be statistically distinguished from each other, and highest signatures were found in SSDF Opheliidae, P/S Nephtyidae and SDF Sphaerodoridae polychaetes. In NOW the signatures of SDF were lower than those of SSDF and P/S, which suggests that the abundant fresh OM maintains a more traditional separation of trophic positions of different feeding guilds, as was also shown by the increased trophic diversity at this site. Indeed, the SDF in NOW have significantly lower signatures than in LS, further highlighting the differences between the sites. In terms of individual taxa, the δ^15^N signatures sometimes contradicted the original trophic position assumptions. Most notably, some animals generally classified as predator-scavengers had lower than expected δ^15^N signatures. Nereis sp. polychaetes in LS occupied trophic level 1.6, and members of the family Lumbrineridae placed on TL1.8 (LS) and 2.1 (NOW). Especially under food limitation, the lumbrinerids can be considered facultative predators that can occasionally engage in deposit feeding [129], which could explain the fairly low δ^15^N signatures. On the other hand, the signatures of opheliids were surprisingly high. Previous fatty acid content analysis has revealed a high amount of zooplankton biomarker 20:1(n-11) in Opheliidae individuals [137]. This has previously been attributed to ingestion of deep sea copepod remains [137,138] and is therefore evidence of a omnivorous/carnivorous diet (Drazen et al. 2008b), even though the family is generally regarded as SSDF. This could therefore explain the high enrichment of opheliids found in this study, especially as more zooplankton remains and faeces is included in the sinking POM later in the summer [69]. The isotopic signatures therefore revealed more detailed site specific information on the actual trophic position of these taxa, compared to their feeding guild designations. Some of the assumptions on feeding methods of the deep sea benthic fauna might thus need to be called into question, as switching between feeding modes and flexible trophic positions seem common [139]. This study suggests that the Arctic benthic ecosystems thrive by generalist feeding on the available food items, and sites with high OM flux to the seafloor can have simpler food webs than would otherwise be expected. It should however be noted that methodological constrains of stable isotope analysis related to sampling timing are inevitably reflected in the current results, and an experimental approach directly comparing ice algae and phytoplankton uptake rates by benthos would ultimately establish faunal dependence on different types of OM.

## Conclusion

This study shows that both North Water Polynya and Lancaster Sound Polynya are among the most significant benthic macroinfaunal hotspots in the Arctic Ocean, where the macrofaunal communities thrive on a diet consisting mainly of phytoplankton food, opportunistically supplemented with ice algae. Contrary to the concerns that a switch from ice algae to phytoplankton diet would reduce the quality of food available to benthos, the high phytoplankton production in both polynyas has led to an increased macrofaunal utilisation of fresh, high quality food, compared to other Arctic regions where macroinfaunal diets mainly consist of refractory OM even at shallow depths. Differences in the total amount of food available was however reflected in the community trophic structure: the higher OM flux in NOW resulted in a shorter food web, with an even number of consumers placing on TL 1 and TL2. The different feeding guilds were also distinguishable by their δ^15^N signatures, showing that the abundant food allowed for selective feeding, maintaining trophic niche separation. In LS, the food web was longer, and the community had a larger proportion of higher consumers. Most fauna appeared to be facultative feeders, which resulted in lower trophic diversity compared to NOW. Based on the results here, the overall increase in phytoplankton primary production in the future could support highly abundant benthic macroinfaunal communities, and sites receiving less OM can establish thriving communities through facultative feeding and efficient recycling of OM within complex food webs. As the creation of such biological hotspots is facilitated by the elongated open water period, it is not beyond reason to suggest that Arctic benthic communities could benefit from the future changes in ice regime and the consequent shift from ice algae to phytoplankton system.

## Supporting information

S1 Table The relative contribution of pelagic POM (a proxy for phytoplankton) and ice algae in macroinfaunal taxa and feeding guild diets based on tissue δ^13^C and δ^15^N measurements in North Water Polynya (NOW) and Lancaster Sound (LS) obtained using SIAR model. P/S = predator/scavenger, SSDF = subsurface deposit feeder, SDF = surface deposit feeder.
(DOCX)
