# Supplementary material for: Benthic macroinfaunal community structure, resource utilisation and trophic relationships in two Canadian Arctic Archipelago polynyas
Source: PLoS One. 2017 Aug 29;12(8):e0183034. doi: 10.1371/journal.pone.0183034 (PMC5574606; doi:10.1371/journal.pone.0183034)
Supplement: S1 Table — P/S = predator/scavenger, SSDF = subsurface deposit feeder, SDF = surface deposit feeder. (DOCX) [file pone.0183034.s001.docx]

|  |  | **NOW** | | **LS** | |
| --- | --- | --- | --- | --- | --- |
|  | **Taxa** | **POM** | **Ice algae** | **POM** | **Ice algae** |
| Polychaeta | Ampharetidae | 0.791 | 0.209 | 0.764 | 0.236 |
| Polychaeta | Lumbrineridae | 0.834 | 0.166 | 0.521 | 0.479 |
| Polychaeta | *Nephtys* sp. | 0.924 | 0.076 | 0.804 | 0.196 |
| Polychaeta | Opheliidae | 0.949 | 0.051 | 0.948 | 0.052 |
| Polychaeta | *Cossura* sp. | 0.597 | 0.403 | 0.875 | 0.125 |
| Polychaeta | Spionidae | 0.769 | 0.231 | 0.467 | 0.533 |
| Polychaeta | Maldanidae | 0.909 | 0.091 | 0.524 | 0.476 |
| Polychaeta | *Asychis* sp. |  |  | 0.564 | 0.436 |
| Polychaeta | *Maldane* sp*.* |  |  | 0.641 | 0.359 |
| Polychaeta | *Spiochaetopterus* sp. |  |  | 0.758 | 0.242 |
| Polychaeta | *Nereis* sp. |  |  | 0.831 | 0.169 |
| Polychaeta | *Scoloplos* sp. |  |  | 0.777 | 0.223 |
| Polychaeta | Sphaerodoridae |  |  | 0.826 | 0.174 |
| Polychaeta | *Sphaerodoropsis* sp. |  |  | 0.921 | 0.079 |
| Polychaeta | *Sphaerodorum* sp. |  |  | 0.543 | 0.457 |
| Polychaeta | Capitellidae | 0.682 | 0.318 |  |  |
| Polychaeta | Cirratulidae | 0.585 | 0.415 |  |  |
| Cumacea | Diastylidae | 0.727 | 0.273 | 0.603 | 0.397 |
| Cumacea | *Eudorellopsis* sp. | 0.668 | 0.332 |  |  |
|  | Bivalvia | 0.591 | 0.409 | 0.491 | 0.509 |
|  | Sipuncula |  |  | 0.513 | 0.487 |
|  | Actiniaria | 0.931 | 0.069 |  |  |
|  | Echinodermata | 0.932 | 0.068 |  |  |
| P/S |  | 0.751 | 0.249 | 0.614 | 0.386 |
| SSDF |  | 0.729 | 0.271 | 0.795 | 0.205 |
| SDF |  | 0.591 | 0.409 | 0.667 | 0.333 |
